# Rules railroad: Syntax-inspired diagrams for visualizing and understanding rule-based model specifications

**DOI:** 10.1371/journal.pcbi.1014121

**Published:** 2026-03-25

**Authors:** Reesha J. Patel, Michael L. Blinov

**Affiliations:** Center for Cell Analysis and Modeling, University of Connecticut Health Center, Farmington, Connecticut, United States of America; University of Auckland, NEW ZEALAND

## Abstract

Rule-based modeling provides a powerful framework for describing and simulating biochemical systems composed of multi-site molecules and multi-molecular species. By encoding molecular interactions as rules rather than enumerating all possible species, this approach naturally accounts for the combinatorial complexity of connectivity within chemical species. Despite these advantages, visualization of such models remains challenging. Existing approaches, such as contact maps, give a high-level overview of possible sites and interactions but lack explicit representation of dynamic processes, while traditional rule cartoons split reactants and products across a reaction arrow, separating molecular context from transformation. We introduce **Rules Railroad (RRR) diagrams**, a novel diagrammatic representation of rule-based model specification. Each RRR diagram encapsulates a single rule as a continuous flow diagram with embedded actions, including binding, unbinding, and state changes. Inspired by classical railroad (syntax) diagrams used to represent formal grammars, RRR diagrams encode both the structural context and the transformations of a rule in a unified format, more compact compared to a classical visualization approach of presenting a single rule as a reactant-product pair. This integration reduces ambiguity, enhances readability, and provides a systematic, human- and machine-readable visualization of any rule-based system. RRR diagrams are precise, and suitable for debugging, communication, and education.

## Author summary

Biological systems often depend on molecules that can exist in many different forms and interact in complex ways. Rule-based modeling is a powerful method for describing and simulating these interactions, but current ways of visualizing rules are either show only some details or split across separate before-and-after diagrams. To address this, we introduce Rules Railroad (RRR) diagrams, a new way of representing rule-based models. Inspired by the railroad diagrams used in computer science to explain programming languages, RRR diagrams combine the *context* of a molecular interaction and the *changes* it causes into a single continuous diagram. This makes them compact, precise, and easy to read.

RRR diagrams help modelers check their rules, debug complex systems, and share ideas with others. They make the logic of molecular interactions more accessible to experimental scientists, students, and collaborators without computational backgrounds. By drawing parallels with circuits in engineering and flows in fluid dynamics, RRR diagrams provide an intuitive map of how biological components interact and change.

## Introduction

Biomolecules such as proteins typically contain multiple functional components, such as phosphorylation and binding sites like tyrosines and SH2/PTB domains. Importantly, interactions among biomolecules are among these binding sites and are often dependent on site-specific molecular details, such as tyrosine phosphorylation [1–5].

Rule-based modeling is a formal approach for describing biochemical systems in which molecular species possess multiple binding sites and internal states [6,7]. It is particularly well suited for modeling systems with combinatorial complexity, such as cell signaling networks, where a small number of molecule types can form a vast number of distinct complexes through various interactions and modifications [8–10].

Instead of explicitly listing every possible species and reaction—as required in traditional reaction-based models—rule-based modeling defines a system through a set of reaction rules. Each rule acts as a template that specifies reactant patterns (partial molecular structures or features that determine which species the rule can act on), product patterns (the result of applying the rule, which may differ from the reactants in terms of site connectivity, e.g., new bonds formed or broken, or internal state, e.g., phosphorylation, activation), and kinetic laws (quantitative expressions, e.g., mass-action rates, that determine the propensity or rate at which the rule applies when its pattern matches a set of molecules).

For example, a rule might specify that: “*A Grb2 molecule with unbound SH2 and SH3 domains can bind via its SH2 domain to a phosphorylated Y1068 site on an EGFR molecule. Other domains on EGFR may be in any state and need not be involved in this interaction*.” This single rule can apply to a wide variety of species—EGFR monomers, dimers, or higher-order complexes—as long as the required features (the phosphorylated Y1068 and unbound SH2) are present. This generalization is the core strength of rule-based modeling: it avoids explicitly enumerating all species and reactions by leveraging pattern-based specification.

Reactant and product patterns are typically described using a graph-like formalism, where molecular sites are nodes, and binding between sites creates an edge [11]. A rule transforms the reactant graph into the product graph by adding or removing bonds, changing internal states (e.g., from “unphosphorylated” to “phosphorylated”), and creating or deleting molecules (e.g., for synthesis or degradation events). These transformations are applied to any species that match the reactant pattern, and the rule can generate multiple distinct reactions during simulation or network expansion.

In addition to defining species and reactions, rule-based models often include observables—formal expressions that define measurable quantities or subsets of species of interest during simulation. Observables can be used to track the concentration of specific molecular patterns or the presence of a particular phosphorylation state, or to measure the number of complexes involving a certain molecule. Observables are defined similarly to reactant patterns, but they do not perform transformations. Instead, they match species during simulation and aggregate them into measurable outputs. For instance, an observable may count all EGFR molecules with phosphorylated Y1068 sites, regardless of the rest of the molecular context. This abstraction enables modelers to align simulations with experimental readouts, such as western blot intensities or fluorescence levels, which typically report on aggregate molecular features rather than individual species.

This modeling and simulation technique is implemented in various popular software tools, such as BioNetGen [12,13], NFsim [14], Virtual Cell [15–17], SmolDyn [18], PySB [19], Kappa [20,21]), Simmune [22], rxncon [23]. Numerous rule-based models are published annually (just in 2025, there are at least three models implemented in BioNetGen, by [24–26], and the accompanying code (such as in BioNetGen Language, BNGL) for these models is typically included in supplementary materials.

While rule-based modeling provides a scalable and expressive framework for describing biochemical systems with complex molecular interactions and modifications, visualizing and interpreting rule-based models remains a significant challenge, especially as models grow in size and complexity. There are several complimentary approaches to visualization of rule-based models.

Cartoon descriptions provide visualizations of the reactant and product patterns for each rule, depicting reactants and products on either side of a reaction arrow, detailing both changes in reactions and their context. Cartoon visualization was first used to describe the first rule-based modeling of receptor-initiated signaling in immunology complicated by multiple trans- phosphorylation events [9,27]. Such cartoons were used in the first papers that described the rule-based software BioNetGen [12] and method [6]. Cartoons were used in almost all research publications using rule-based modeling and were formalized in the VCell modeling and simulation framework [16,17]. Finally, web visualization in the form of rules was developed by Liguori-Bills & Blinov, 2024 [28].

However, cartoon visualization scales linearly with the number of rules, and may be difficult to follow if the rule involves several different actions, such as simultaneous breaking and forming several bonds. The proposed RRR diagram visualization extends and improves cartoon visualization, still describing each individual rule as a single cartoon, although in a more structured and compact way.

There are approaches that attempt to provide a compact visualization of the complete rule-based model. Contact maps provide a compact representation of molecular interactions by highlighting the components (e.g., binding sites, phosphorylation sites) and their interactions in one map. Contact maps are well-suited for large systems with many rules. However, they hide the context of binary interactions, even in the more complicated extended contact map form [29]. Rule influence diagrams [30], atom-rule graphs [31], modular representation of reactions contingency extension *rxncon* [23,32], and the Simmune NetworkViewer [33,34], aim to illustrate the relationships and influences among rules or molecular features. The NetworkViewer, for example, creates a bipartite graph linking rules to specific reactant/product patterns, closely resembling traditional pathways. However, this approach becomes impractical for models exhibiting combinatorial growth in the number of nodes when molecules have multiple binding configurations. Molecular Process Diagrams [35] provide another visualization view of the complete model by defining a graph with nodes corresponding to various molecular combinations encountered in reaction rules. However, this approach does not scale well when the rules have a lot of contexts.

To address this challenge, we introduce Rules Railroad (RRR) diagrams—an intuitive and precise visualization method designed to enhance both human comprehension and machine processability of rule-based models. RRR diagrams embed both the reactant context and the rule’s action within a single visual structure. Each RRR diagram represents the full molecular pattern to which a rule applies, visually integrates modifications (e.g., bond formation, state changes), clearly distinguishes between required context and changes performed by the rule. We follow conventions introduced by the NFsim input file [14], which for each rule lists actions to be performed on the species selected by the rule. However, these conventions were never visualized before.

RRR diagrams are inspired by railroad (syntax) diagrams used in formal language theory, where diagrams define valid strings in a grammar. Similarly, each RRR diagram can be interpreted as a pattern recognizer that defines the set of species a rule applies to and the transformations it induces. This analogy reinforces the generative nature of reaction rules: just as grammars generate strings, rules generate reactions.

By providing a visually structured, color-agnostic, and annotation-rich format, RRR diagrams make rule-based models more accessible to interdisciplinary audiences, including experimental biologists and students. They support effective communication of complex rule-based logic in publications, presentations, and teaching materials. Moreover, RRR may facilitate model debugging and validation, by helping users visually verify that rules behave as intended.

RRR diagrams are designed to be machine-readable and can be generated directly from formal rule definitions (e.g., BNGL). Our implementation includes software that parses BNGL rules, identifies relevant molecular patterns, encodes the diagram structure, and outputs SVG visualizations that are human-readable.

In the discussion section, we will describe how RRR diagrams may link rule-based modeling to other scientific and engineering domains, such as computer science and language theory, electrical engineering, and fluid dynamics.

## Results

### Railroad diagrams

**Fig 1:**
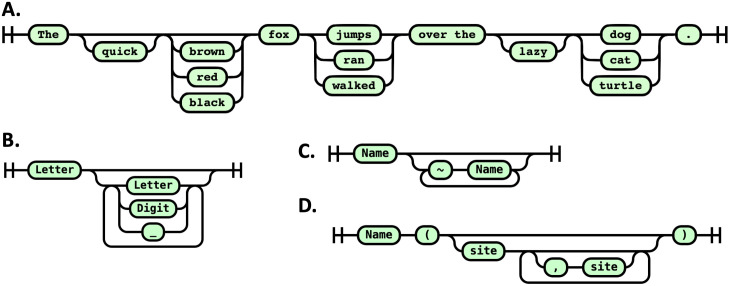
Classical syntax (railroad) diagrams. **A**. Syntax diagram defining a language of all sentences of the form “The {quick, —} {—, brown, red, black} fox {jumps, ran, walked} over the {—, lazy} {dog, cat, turtle}.” Here, each bracketed group offers choices, and “—” means the element can be omitted. This gives: 2 × 4 × 3 × 2 × 3 = 144 possible sentences, such as: “The quick fox walked over the lazy cat.” or “The brown fox ran over the dog.” **B**. The railroad diagram for BNGL syntax. A Name is any sequence of letters, digits, or underscores, beginning with a letter. **C**. A site is a name optionally followed by one or more states that are defined as a name preceded by the tilde symbol. **D**. A molecule is a name optionally followed by a parenthesized, comma-separated list of sites.

In simple terms, a railroad diagram is a visual representation of a formal grammar [36]. It resembles a flowchart and shows how valid sentences in a language can be constructed. Each part of the sentence is represented as a path that can be followed from left to right. To be part of the language, a word (or sentence) must follow a valid path through the diagram—from the entry point to the exit. The diagram illustrates all possible valid paths, as illustrated in Fig 1A.

### BNGL language

The description of a biological system in rule-based languages such as BNGL (BioNetGen Language) consists of symbolic statements that define molecules, species, observables, reaction rules, and other components necessary to fully represent and simulate the system. The structure of a BNGL input file adheres to a formal grammar—a set of production rules that define all valid strings in BNGL.

An example of the grammar for BNGL, written in Extended Backus–Naur Form (EBNF), is shown below. This example represents a simplified subset of the full BNGL grammar described in Harris et al. (2016) [13], with some features such as compartments and pattern matching omitted for clarity:

Name = Letter, {Letter | Digit | “_”};

State = “~”, Name;

Site = Name, [{“~”, State}};

Molecule = Name, [“(“, [Site, {”,”,Site}],”)”];

This formal description is difficult to follow, so railroad diagrams provide an easier way to follow this grammar (Fig 1B).

### Case Study: EGFR signaling as a model system in RRR diagrams

In our project, we use railroad diagrams to visually represent rule-based models, extending the classical railroad formalism by introducing additional elements. Our Rules Railroad (RRR) diagrams do not depict BNGL strings directly, but instead illustrate the composition and connectivity of multi-site molecules, as well as the rules governing their interactions.

In the examples below, we focus on the epidermal growth factor receptor (EGFR)—a well-studied and extensively modeled system that plays a critical role in cell signaling and is implicated in pathological conditions such as breast cancer. EGFR is an ideal case for exploring rule-based modeling, as numerous models have been developed for this system, and its molecular mechanisms are well characterized [e.g., 8,37–39].

The EGFR protein consists of three main domains: an extracellular domain that binds ligands such as EGF, a transmembrane domain that anchors the receptor in the membrane and facilitates lateral diffusion and dimerization, and an intracellular domain containing multiple tyrosine residues, which can be phosphorylated and serve as docking sites for adapter proteins that initiate downstream signaling cascades. These domains contribute modularly to signaling and can be described independently in rule-based models, though they function together in the biological system.

Importantly, EGFR receptors can form higher-order clusters through pre-formed dimers via transmembrane interactions, or ligand-induced dimerization through the extracellular domain. The intracellular domain contains around ten tyrosine residues, each potentially capable of binding proteins with SH2 or PTB domains. However, due to the complexity of the full system, rule-based models typically include only a subset of these tyrosines—selected based on which protein-protein interactions are relevant to the modeling goals. The same principle applies to adapter proteins: only the functional domains involved in the modeled interactions are explicitly represented.

In our examples below, we consider a simplified subset of EGFR-mediated signaling, following Blinov et al. (2006a) [8]. The EGFR molecule is modeled with one extracellular domain labeled “l”, one transmembrane domain labeled “r”, and two intracellular tyrosine sites: Y1068 and Y1148, each of which can exist in two states—unphosphorylated or phosphorylated. The Grb2 adapter protein is modeled with two functional domains: one SH2 domain that binds phosphorylated tyrosines, and one SH3 domain, which may participate in additional interactions. This simplified system allows us to illustrate key features of rule-based modeling using Rules Railroad (RRR) diagrams, while keeping the biological context grounded and interpretable.

### RRR visualization

We aim to provide a representation that is unambiguous, visually clear, and simultaneously human readable and machine-processable. Colors in our diagrams are optional and used solely to enhance human readability. They are not required for correct interpretation. All elements of the notation are color-agnostic, ensuring that the diagrams can be fully understood and processed without relying on color cues. Our goal is not to represent BNGL grammar elements such as brackets or tilde symbols, but rather the core components relevant to interaction rules: the molecule name, its binding sites, and the possible states of those sites.

### RRR representation of molecules

**Fig 2:**
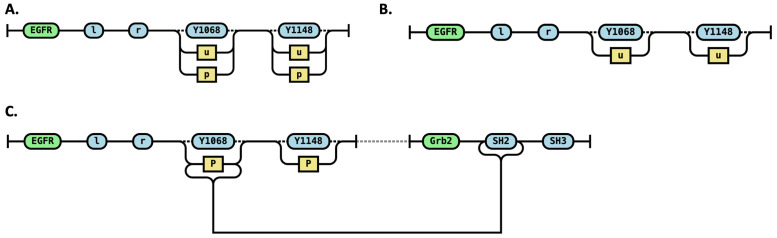
RRR diagrams representing molecules, species, and complexes. **A.** EGFR molecule with 4 sites l, r, Y1068 and Y1148, with the last two being in two possible states each - unphosphorylated “u” and phosphorylated “p”. **B**. An EGFR species represented by an EGFR molecule with sites Y1068 and Y1148 both in the unphosphorylated (“Y”) state. **C.** A species consisting of an EGFR molecule connected to a Grb2 molecule via a bond between the phosphorylated (“p”) state of the Y1068 site on EGFR and the SH2 site on Grb2.

To represent molecules, we follow railroad diagram conventions with minor modifications (Fig 2A).

The objective is to preserve the railroad diagram’s convention of enumerating all possible forms of a molecule—in this case, four forms, based on two sites (Y1068 and Y1148), each of which can exist in two distinct states. The site names are shown as dashed-line annotations in the diagram, as they are not explicitly part of the enumeration of molecular forms.

### RRR representation of species

In RRR diagrams, a chemical species corresponds to a single valid “word,” so a species consisting of a single molecule is represented by one continuous path (Fig 2B). However, a chemical species may also consist of multiple molecules connected by site-specific interactions, called bonds. Following railroad diagram conventions, we visually represent these bonds as smooth connections between specific endpoints (bound states of sites) on different molecules. These connections are analogous to paths in traditional railroad diagrams (Fig 2C). Alternatively, these connections can be considered as constraints on the context-free grammar [40].

Additionally, molecules within the same species are connected using dashed lines. These lines indicate connectivity but do not represent actual paths used to enumerate valid strings in the grammar. While dashed lines may appear redundant for species already explicitly connected by bonds, their significance becomes clear when defining observables.

### RRR representation of observables

Observables are critical components in rule-based modeling frameworks. They allow researchers to define groups of chemical species that share common structural or functional properties, comparing simulation outcomes with experimental observations. Typically, observables represent subsets of species characterized by specific molecular patterns or states.

**Fig 3:**
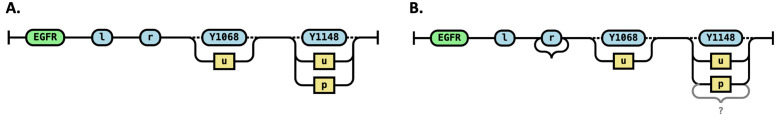
Observables. **A**. An observable describing all species that include only EGFR molecule with site Y1068 in phosphorylated state. **B**. An observable describing all species that include EGFR molecule bound at site “r” and optionally bound at site “Y1148”.

In RRR diagrams, some observables can be conveniently represented using classical railroad diagram conventions, which enumerate all possible molecular forms consistent with certain criteria. For example, consider an observable defined by an EGFR molecule in which the Y1068 site is phosphorylated (“p”), while the Y1148 site can exist either in an unphosphorylated (“u”) or phosphorylated (“p”) state (Fig 3A). This specification results in two distinct chemical species grouped within a single observable. The railroad diagram below clearly illustrates this scenario, explicitly enumerating both possible molecular configurations, thus providing visual clarity and ease in defining and interpreting observables within the BioNetGen framework.

However, observables can often have more complex specifications. They may not only describe individual molecular states but can also explicitly permit or require connectivity between molecules through specific sites in specific states. Thus, a RRR diagram clearly distinguishes two scenarios: mandatory connectivity and optional connectivity. In the first case, a site (or set of sites in specified states) must be bound to another molecule. Such a constraint ensures that only species featuring a particular bond at that site are included in the observable. For optional connectivity, a site (or set of sites in specified states) may either be bound or remain unbound. Species are included in the observable regardless of whether this bond is present.

For example, in Fig 3B, the EGFR site **r** is shown as requiring a bond—it must be connected to another molecule. Meanwhile, the site Y1148 is illustrated as optionally connected; it may form a bond in either the unphosphorylated (“Y”) or phosphorylated (“pY”) states. This visual representation precisely captures both mandatory and optional connectivity, clearly defining which chemical species will be considered part of the observable within the BioNetGen simulation framework. In agreement with railroad diagrams conventions, we define these mandatory or optional bonds as required (solid traversal) or optional (grey traversal with a question mark) paths.

### RRR representation of reaction rules

#### RRR representation of binding and unbinding interactions.

In RRR diagrams, binding and unbinding interactions are represented using standardized visual elements that specify the conditions under which two molecular sites form or break a bond. These diagrams capture both the structural context and the directionality of the interaction, allowing for unambiguous interpretation of both irreversible and reversible reactions.

Reaction rule**s** serve as descriptors of individual reactions that act on chemical species. Each rule is defined by two components: (1) the structural or state features of the species it applies to, and (2) the specific transformation it performs, such as binding, unbinding, or modification of a site.

For example, consider the following rule:

*A Grb2 molecule with unbound SH2 and SH3 sites can bind via its SH2 site to the phosphorylated Y1068 site of an EGFR molecule. Other sites on EGFR do not influence this interaction—they may be in any allowable state or may be bound to other sites on other molecules.*

This rule captures a specific biochemical event while abstracting away irrelevant molecular context, enabling compact yet precise modeling of complex signaling systems. This single rule represents multiple concrete reactions, such as binding of free Grb2 to an unbound EGFR monomer, an EGFR dimer, or an EGFR molecule already part of a larger complex with other ligands or proteins, as long as the EGFR contains an unbound, phosphorylated Y1068 site. The rule thus compactly encodes a large class of potential reactions, reflecting one of the core advantages of rule-based modeling: the ability to generalize over molecular context while preserving mechanistic detail.

**Fig 4:**
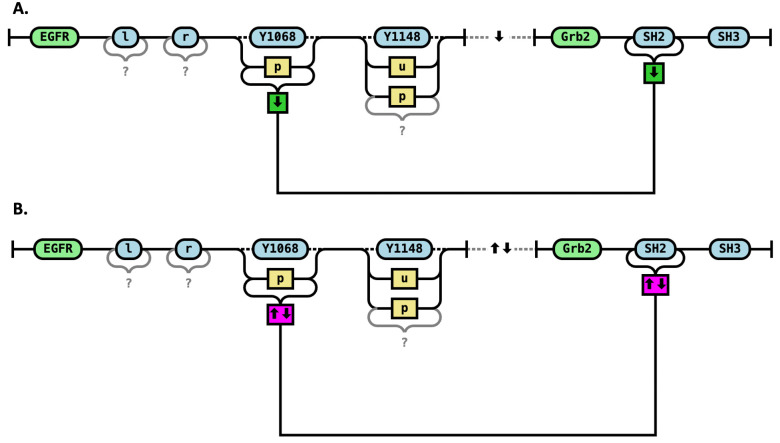
Irreversible rules for binding (A) and unbinding (B) of the SH2 domain of Grb2 to the Y1068 site of EGFR in the phosphorylated (“p”) state. The SH3 site of Grb2 must be unbound for the interaction to occur. Other sites on EGFR are not constrained—they may be in any allowable state and may be either bound or unbound.

To convey the rule clearly, we use both colors and symbols (Fig 4). Colors are included for visual clarity—green indicates bond formation (“go”) and red indicates bond destruction (“stop”). Symbols inside boxes are used to encode the rule in a machine-readable and unambiguous form, suitable for automated analysis and processing. Specifically, a green box (accompanied by a downward arrow) indicates that the rule results in the creation of a bond between the specified sites. This visual language allows us to compactly and precisely represent complex rule-based interactions in a way that is accessible to both humans and software tools.

The color of boxes provides an intuitive and precise way to represent the transformation, while the direction of the arrow is machine-readable and color-agnostic. Together, these elements allow the RRR diagram to clearly distinguish between rules that form bonds and those that break them, supporting both human readability and machine interpretation.

**Fig 5:**
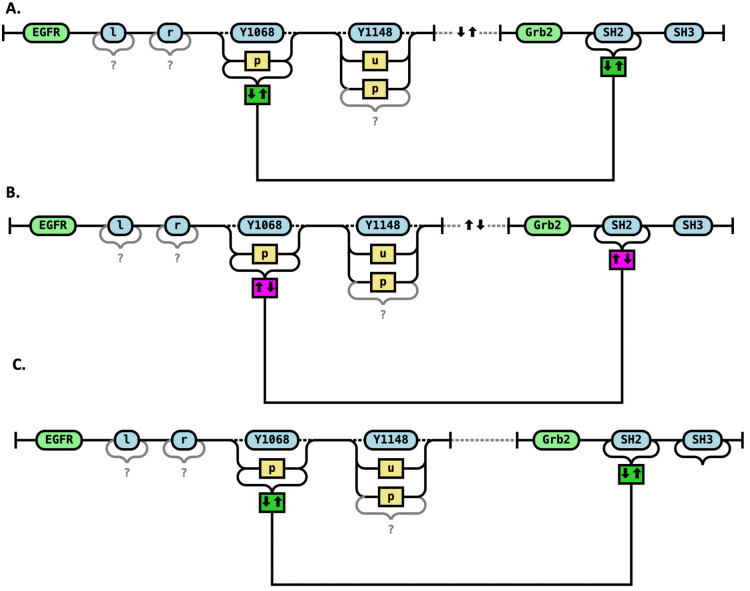
Reversible rules. Binding (A) and unbinding (B) of the SH2 domain of Grb2 to the Y1068 site of EGFR in the phosphorylated (“p”) state. The first arrow specifies the direction of the forward reaction: a downward arrow indicates that the forward rule creates a bond, while an upward arrow indicates that the forward rule breaks a bond. The color of the box reflects the nature of the forward direction: a green box denotes that the forward rule creates a bond, while a red box denotes that the forward rule destroys a bond. **C.** Intramolecular binding. Grb2 is already part of a complex with EGFR (indicated by a dashed line), and it binds/unbinds via its SH3 domain (shown by a solid bond). The rule shown is reversible and establishes a new bond between the SH2 domain of Grb2 and the phosphorylated Y1068 site of EGFR.

However, some rules are reversible, meaning they can proceed in both forward and reverse directions. In our RRR diagrams, we represent this reversibility precisely using a combination of colors and symbols. Reversibility is indicated by a double arrow inside the box (Fig 5). This dual encoding provides a clear and unambiguous representation of reversible rules, allowing users to immediately identify the direction and effect of both the forward and reverse reactions.

### RRR representation of intramolecular vs intermolecular interactions

In Rules Railroad (RRR) diagrams, we distinguish between intramolecular and intermolecular interactions—an important distinction in rule-based modeling, as it affects both the interpretation of reaction rules and their kinetic behavior.

Intramolecular interactions occur within the same molecule or molecular complex. These often involve sites that are part of the same molecular species and are already connected by bonds, directly or indirectly. Such interactions are influenced by spatial proximity and may proceed at faster rates due to the confined context.

Intermolecular interactions, in contrast, occur between separate molecular species that are not connected prior to the interaction. These events depend on the diffusion and encounter of the participating species and typically have different kinetic parameters.

In RRR diagrams intramolecular interactions are depicted using dashed lines to indicate that the interacting molecules or sites are already part of the same complex. Intermolecular interactions lack such dashed lines, indicating that the interaction involves association of previously unconnected species. This visual distinction allows modelers and readers to immediately recognize the context in which a rule operates, improving both interpretability and model accuracy.

In the earlier examples, Grb2 was not associated with EGFR before the reaction rule was applied—the interaction involved the initial binding of a free Grb2 molecule to the EGFR receptor. However, Grb2 may already be part of a molecular complex that includes EGFR, connected through a chain of adapter proteins. In this case, Grb2 can still bind to the phosphorylated Y1068 site of the same EGFR molecule. Because Grb2 is already near the receptor, the effective local concentration is higher, and this intra-complex interaction is expected to have a higher reaction rate than the association of Grb2 with an EGFR molecule from a different complex.

Fig 5C illustrates such a scenario, where two molecules are already in a complex and a new bond is formed. Since the precise path of connectivity between Grb2 and EGFR is unknown in this case, all other bonds on EGFR are marked as potential—denoted with question marks—indicating that they may or may not exist but are not constrained by the rule. This representation allows for flexible matching of complex molecular contexts while preserving key mechanistic details.

### RRR representation of state changes

**Fig 6:**
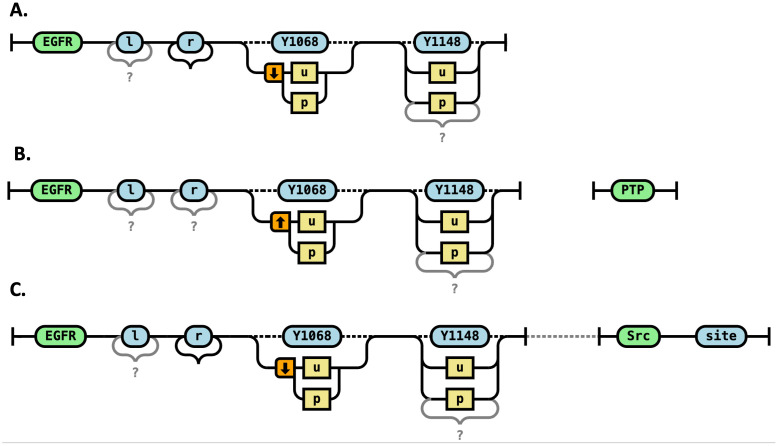
State changes and catalysis. **A.** Transphosphorylation of EGFR at the Y1068 site of the EGFR receptor. This reaction has two key requirements: (1) The receptor must be dimerized, specifically through an explicit bond at the “r” site, which corresponds to the transmembrane domain. This bond ensures that the two EGFR molecules are part of the same dimeric complex, enabling one receptor to phosphorylate the Y1068 site of its partner. (2) The Y1068 site on the target receptor must be unbound, allowing it to undergo phosphorylation without interference from existing interactions. **B.** Dephosphorylation reaction catalyzed by PTP not bound to receptor complex. In this case PTP molecule is not necessary binding to any other molecule, so it may lack any sites. **C.** Phosphorylation reaction of EGFR dimer catalyzed by Src kinase that is a part of the receptor complex. Note that to be part of a complex, at least one site of Src is required.

In RRR diagrams, we represent state changes using distinct visual elements that highlight how molecular components are modified. State changes represent transitions in the internal state of a molecular site—such as phosphorylation, dephosphorylation, activation, or conformational switching. In RRR diagrams, these changes are depicted by a before-and-after pair of states shown along a continuous path, where the site name is preserved, but the state label changes from one value to another (e.g., from “u” to “p”). A symbol (a vertical arrow) is included in the orange box to indicate the direction of the transition (Fig 6C). This representation makes it visually clear which site is undergoing a state transformation and what the outcome of the rule is.

### RRR representation of catalysts

Catalysts are molecular entities that facilitate a transformation—such as a state change or bond formation—without themselves undergoing any modification. In rule-based modeling, catalysts are typically included in the reactant and product patterns unchanged.

In RRR diagrams, catalysts can be represented in two distinct ways, depending on their mode of involvement in the reaction. It can be shown separately (Fig 6B), if it is not directly connected to the target of the reaction. This is appropriate when the catalyst’s presence (e.g., PTP phosphatase for dephosphorylation) influences the reaction but does not require explicit binding to the receptor in the model. In such cases, the catalyst acts globally, and its concentration affects reaction rates without needing to form a specific complex. It also can be shown connected via dashed line (Fig 6C), if the catalyst is part of the same molecular complex—for example, bound to another receptor or scaffold protein—but is not directly interacting with the target molecule, it can be shown as connected through dashed lines. This indicates co-complex membership without implying direct binding to the site being modified. The catalyst’s presence in the complex is necessary for the reaction to proceed, but it is not required to be bound to the specific molecule undergoing the state change.

This flexible representation allows RRR diagrams to capture both explicit and contextual catalytic roles while maintaining clarity and precision.

### RRR changes in molecular composition

**Fig 7:**
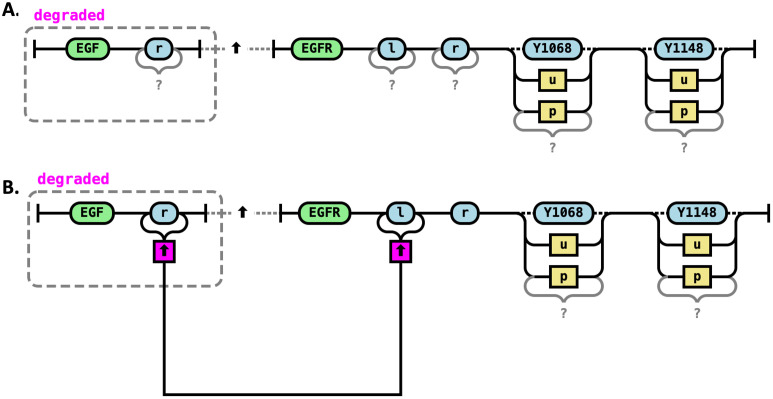
Changes in molecular composition. A. pro-EGF is processed to synthesize EGF. This transformation involves removing the pro-domain and producing a new molecular species, EGF, which can go on to interact with EGFR receptors. **B.** Oversimplified rule for degradation of EGF molecules in a complex. It involves removing a bond and a dashed line between the molecules.

Reaction rules can also specify the synthesis of new molecules or the degradation of existing ones. In RRR diagrams, these events are indicated using dashed boxes drawn around the relevant molecular components (Fig 7A). Components to be degraded are enclosed in dashed boxes, labeled “degraded” in red. Components to be synthesized are enclosed in dashed boxes, labeled “synthesized” in green. While colors are optional, they significantly improve readability and allow for rapid visual comprehension of the rule’s effect.

A reaction rule may also specify the degradation of a molecule that is part of a larger complex. Note that if the molecule is explicitly bound to another molecule via a defined bond, then the degradation rule must also explicitly sever that bond (Fig 7B). This ensures that the resulting complex remains structurally valid and that no dangling references to the degraded molecule remain.

## Discussion

By unifying context, transformation, and visualization in a formal yet accessible format, RRR diagrams enhance both the usability and communicability of rule-based models—making them a valuable addition to the modeling toolkit for systems biology and beyond. They provide a different angle for viewing the reaction rules. The webpage https://rulesrailroad.github.io/examples.html has traditional rules cartoons created with bnglViz (https://bnglviz.github.io/; [28]) and RRR diagrams created with RulesRailRoad (https://rulesrailroad.github.io/) for the same set of rule-based models. Many of the same models are visualized as atom-graphs in the paper by Sekar et al. (2017) [31] and as Molecular Process Diagrams in the paper by Vasilescu et al. (2025) [35]. Screenshots of the full set of rules alongside birds-eye view of these models are available at https://github.com/vcellmike/MolecularProcessDiagram.

Contact maps provide a static, high-level schema of the components and sites that exist and can potentially interact. RRR Diagrams specify the logic of each rule, combining both structure and action, thus suitable for understanding how a rule modifies a system, not just what elements exist. Contact maps are like a blueprint of system connectivity, while RRR diagrams show how system elements are connected and reconfigured in each step.

The authors are particularly intrigued by the railroad-diagram formalism because it suggests the existence of continuous paths across molecular components. In this sense, we do not view railroad diagrams solely as a grammatical construct, but rather as a representational framework that may invite analogies to “flow-based” sciences, such as electrical circuit theory or fluid dynamics, where system behavior is understood in terms of constrained pathways and connectivity. Specifically, RRR diagrams can be used as a visual and semantic bridge to electrical circuit analogies. Indeed, both electrical circuits and rule-based models operate on networks of interacting elements that are governed by local interaction rules (e.g., Ohm’s law, state changes) and follow conservation laws (e.g., current conservation, mass conservation). Site in a rule-based model corresponds to a circuit terminal and a connection bond in RRR. Binding/unbinding naturally corresponds to closing/opening of a switch or wire. State change corresponds to toggling a gate. Similarly to electrical circuits, RRR diagrams map to the main concepts in fluid dynamics, where molecules correspond to pipe sections, binding sites correspond to valves or pressure-controlled gates, binding/unbinding correspond to opening/closing valves between pipe sections, and state changes correspond to adjustments in pressure regulated at control valves.

RRR diagrams provide a powerful and intuitive framework for representing rule-based models in biology, addressing key challenges in model interpretation. RRR diagrams unify the structural context and transformative actions of a rule within a single visual structure. This integration helps modelers verify that rules behave as intended and simplifies the process of debugging models. Unlike traditional representations that separate the left-hand and right-hand sides of a rule, RRR diagrams allow for a cohesive depiction of the molecular conditions required for a rule to fire and the changes it enacts. This visual coherence reduces ambiguity and supports the construction of models that are both scalable and logically precise, particularly in systems with combinatorial complexity such as signaling networks and post-translational modifications. It could be possible to extend RRR diagrams to other rule-based languages such as Kappa, as well as SBML-multi standard designed for exchange of rule-based models, although each such extension would require additional considerations specific for each language.

Beyond their use in model building, RRR diagrams can serve as a meaningful representation of biological knowledge [41], showing not only what components are involved but also how their states and connections evolve. As a visualization method, RRR diagrams integrate context and transformation into a linear, railroad-style flow. This format, inspired by syntax diagrams used in language theory, supports clarity and accessibility while avoiding reliance on color or overly complex symbols. The diagrams can be easily annotated for educational or explanatory purposes, making them suitable for inclusion in publications, presentations, and teaching materials. Their structured and flexible format allows interpretation by experienced modelers, students, and biologists without rigorous computational training.

For non-modelers such as experimental biologists or interdisciplinary collaborators, RRR diagrams offer an easy way to introduce rule-based modeling. Their intuitive design lowers the barrier to understanding complex logic by providing a visual map of molecular interactions and changes. RRR help introduce core ideas of rule-based modeling without the need for formal syntax or simulation knowledge.

RRR diagrams also link rule-based modeling to other scientific and engineering domains. Their formal similarity to syntax diagrams aligns them with the rigor of computer science and language theory. In electrical engineering, the representation of rules as flow paths and transformations suggests analogies with circuits, where binding events act like switches and state changes are similar to voltage transitions. Similarly, in fluid dynamics, the diagrams can be viewed as flow networks where molecules represent pipes, and rules function like valves or junctions. We hope that RRR diagrams may facilitate cross-domain thinking, enabling biological models to be analyzed using tools and intuitions from other sciences.

RRR diagrams are not universal tool. They can visualize individual rules, but they do not provide an overall view of the whole model. They do not describe kinetics laws, kinetic rate constants, quantitative parameters, parts related to simulations such as functions, or parts related to rules applications such as the stoichiometry of molecular complexes or “include/exclude” statements.

In summary, Rules Railroad diagrams enhance the representation, interpretation, and communication of rule-based biological models. By providing a compact, accessible, and expressive visualization format, they support the needs of modelers, educators, and interdisciplinary collaborators. Their connections to formal systems in computer science, electrical circuits, and fluid mechanics further demonstrate their versatility and potential as a visual grammar for biological interaction logic.

## Methods

The software is implemented as an embedded JavaScript tool deployed at GitHub Pages and freely available at http://RulesRailRoad.github.io. The code is described in README.MD available at https://github.com/rulesRailRoad/RulesRailRoad.github.io/. This package is designed to convert BioNetGen Language (BNGL) strings into visual railroad diagrams to represent biological networks and reaction mechanisms.

**Fig 8:**
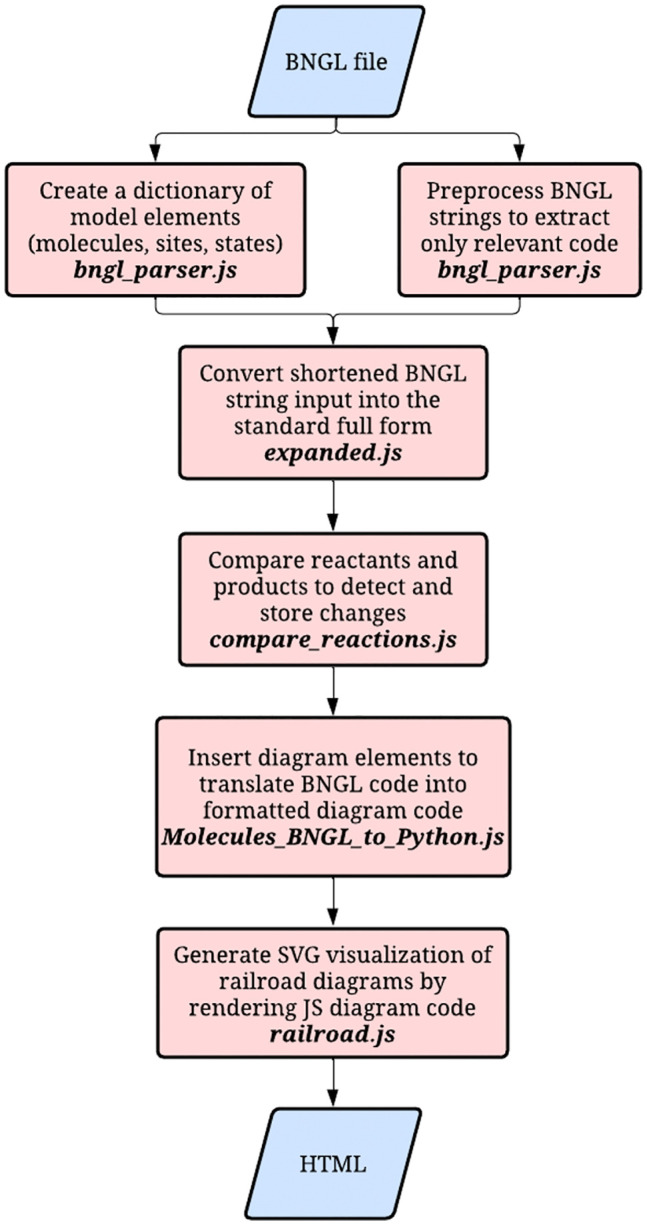
RuleRailRoad package workflow. The bngl_parser.js module preprocesses strings from BNGL code files. expanded.js returns fully expanded BNGL strings and compare_reactions.js finds differences between reactants and products in a BNGL reaction rule. Molecules_BNGL_to_Python.js is designed to take these parsed BNGL strings and translate them into formatted diagram code to be drawn by railroad diagram classes. railroad.js is a railroad-diagram renderer that reads the diagram code and generates the SVG railroad visualization. These diagrams show molecular interactions by highlighting sites, states, bonds, and changes through reactions.

The code consists of multiple integrated JavaScript modules designed to parse, process, and draw BNGL models as railroad diagrams (Fig 8). We provide a series of examples for previously published models and an option to upload and visualize any BNGL file. The original inspiration was provided by Tab Atkins JS + SVG library for drawing railroad syntax diagrams. The Python code was used for prototyping, and then the development was performed in JavaScript.

The visualization is highly customizable, with options to show or hide bond indexes, shorten RRR diagram by omitting molecule names, display or hide comments in BNGL input, and even switch between BNGL-style notations (Molecules-Species) and generic notations (Interacting Agents - Initial Set), if someone will use the tool for non-biological applications.
